# Clinical efficacy of virtual reality for acute procedural pain management: A systematic review and meta-analysis

**DOI:** 10.1371/journal.pone.0200987

**Published:** 2018-07-27

**Authors:** Evelyn Chan, Samantha Foster, Ryan Sambell, Paul Leong

**Affiliations:** 1 Department of Paediatrics, Monash Medical Centre, Clayton, Victoria, Australia; 2 Southern Clinical School, Monash Medical Centre, Clayton, Victoria, Australia; 3 Monash Lung and Sleep, Monash Medical Centre, Clayton, Victoria, Australia; Cedars-Sinai Medical Center, UNITED STATES

## Abstract

**Background:**

Acutely painful procedures are commonplace. Current approaches to pain most often involve pharmacotherapy, however, there is interest in virtual reality (VR) as a non-pharmacological alternative. A methodologically rigorous systematic review and meta-analysis is lacking.

**Methods:**

Following PRISMA guidelines, we searched the Cochrane Library, Ovid MEDLINE, Embase, CINAHL, ERIC, NIHR Centre for Review and Dissemination, Proquest, the System for Information on Grey Literature in Europe and the WHO International Clinical Trials Registry Platform from inception to 5 November 2017. Included studies were randomised with an experimental trial design, included a non-VR control group and examined the efficacy of VR with regards to an acutely painful clinical intervention. Bias was assessed along Cochrane guidelines, with performance bias not assessed due to the non-blindable nature of VR. We extracted summary data for maximal pain score and used standard mean difference DerSimonian-Laird random-effects meta-analysis (RevMan 5.3). This review was prospectively registered (PROSPERO CRD42017058204).

**Findings:**

Of the 12,450 studies identified, 20 studies were eligible for the systematic review. No trials reported in sufficient detail to judge their risk of bias, and 10 studies were at high risk of bias in at least one domain. 16 studies (9 randomised controlled trials, 7 crossover studies) examining 656 individuals were included in quantitative synthesis. Pain scales were heterogenous, but mostly employed 100-point scales. Across all trials, meta-analysis was suggestive of a -0.49 (95%CI -0.83 to -0.41, p = 0.006) standardised mean difference reduction in pain score with VR. However there was a high degree of statistical heterogeneity (χ^2^ p<0.001, I^2^ 81%, 95%CI for I^2^ 70–88%), driven by randomised studies, with substantial clinical heterogeneity.

**Conclusion:**

These data suggest that VR may have a role in acutely painful procedures, however included studies were clinically and statistically heterogenous. Further research is required to validate findings, establish cost efficacy and optimal clinical settings for usage. Future trials should report in accordance with established guidelines.

## Introduction

The management of acute pain related to healthcare interventions remains a major global healthcare challenge[1], existing at the convergence of the consumer-driven desire for patient empowerment and physician-driven desire for better outcomes[2]. For most procedures, pharmacological approaches remain the mainstay although these have significant drawbacks including imprecise titration, narrow therapeutic windows, adverse side effects, the potential for drug misuse and cost[3]. Approaches that avoid pharmacotherapy and associated interventions such as monitoring could therefore be of benefit in a multimodal armentarium[1].

Virtual reality (VR) is a developing technology which has garnered significant lay and medical attention as its cost and accessibility and quality have favourably converged. Briefly, virtual reality is a computer-generated depiction of an immersive environment which can be viewed through a headset[4]. By providing distraction, this approach is hypothesized to reduce pain by pharmacological-sparing means[4].

However, there is no comprehensive, high-quality systematic review that specifically assesses the efficacy of virtual reality on acutely painful healthcare interventions, nor has there been any quantitative data synthesis on this topic. We therefore conducted a systematic review and meta analysis to appraise the quality of published literature and to synthesize data for acute pain scores.

## Methods

### Study selection, data sources and search strategy

We defined VR as an intervention with an immersive, 3D display that excluded the external (real-world) environment. Studies were included if they were published in a peer reviewed journal, examined the effect of VR on an acutely painful clinical intervention and included a pain score as an outcome measure. Studies were excluded if there was no acutely painful clinical intervention, no non-VR control group or non-VR sequence or lacked an experimental design. This review and protocol was prospectively registered on PROSPERO (CRD42017058204).

Following PRISMA guidelines[5], we identified studies through reviews of the Cochrane Library, Ovid MEDLINE (1975–5 November 2017), Embase, CINAHL, ERIC, NIHR Centre for Review and Dissemination and Proquest (PRISMA checklist: S1 Checklist). The search strategy included the terms “virtual reality”, “simulation”, and “pain”: the full strategy is in S1 Appendix. For completeness, we searched the System for Information on Grey Literature in Europe and WHO International Clinical Trials Registry Platform. No language restrictions were applied. Non-English articles were machine translated and screened for inclusion. Automatic de-duplication was performed in EndNote X8.1 (Clarivate Analytics, Philadelphia USA), and manually verified by an author (EC). Citation lists of included studies were hand checked to ensure completeness. Screening was performed by two authors (SF, RS) and disagreements resolved consensus discussion with a third author (EC).

### Data analysis

Summary data was extracted by one author (PL) and confirmed by another author (EC). For parallel group randomised trials (RCTs), the Cochrane risk of bias assessment tool was used[6]. For crossover trials, a published modification of this tool was employed[7]. Two authors (PL, EC) independently assessed risk of bias, with verification by the other two authors (SF, RS). Disagreements were resolved by consensus.

The following information was extracted from each study: first author name, study location, source and number of participants, ethics approval, age, sex, study design, and virtual environment and nature of painful stimulus. The primary outcome was the mean difference in maximum self-rated pain during the healthcare intervention (with and without VR). If the study included interventions other than VR, only data relevant to pain scores with and without VR was extracted. If the study had multiple treatment periods, the first was extracted. If data were not reported in an analysable format, summary measures were reconstructed from published individual patient data, or authors approached. Where data were missing, first authors were contacted twice by e-mail at one-month intervals, and if data were still missing, senior authors were contacted similarly; if authors had moved, attempts were made to contact them at their new institutions.

It was anticipated that crossover trials would pose difficulties and thus employed Elbourne’s “ideal” method (within-individual data)[8]. In brief, correlation coefficient was sought and missing data imputed by Elbourne’s published method[8]. We used standard mean difference (SMD) DerSimonian-Laird random-effects meta-analysis (RevMan 5.3, Copenhagen) to estimate effect size on pain.

Variability within studies is reported in forest plots and incorporated into the meta-analysis (I^2^), and interpreted in accordance with standard guidelines[9]. To quantify uncertainty in the I^2^ statistic, we calculated heterogeneity in I^2^ as recommended[10] using heterogi[11] in Stata 14.2 (College Station, Texas). The calculation requires at least two degrees of freedom.

Risk of bias was assessed but other no methods to account for this were employed. *A priori*, due to the obvious nature of VR, performance bias was not assessed. Detection bias was assessed as high if an unblinded investigator assessed outcomes, low if a blinded observer assessed outcomes and unclear if self-administered instruments were used. Funnel plots were inspected for asymmetry to assess for sources of bias including publication bias[12].

### Role of the funding source

There was no funding source for this study. All authors had full access to data and the corresponding author takes responsibility for the decision to submit to publication.

## Results

**Fig 1:**
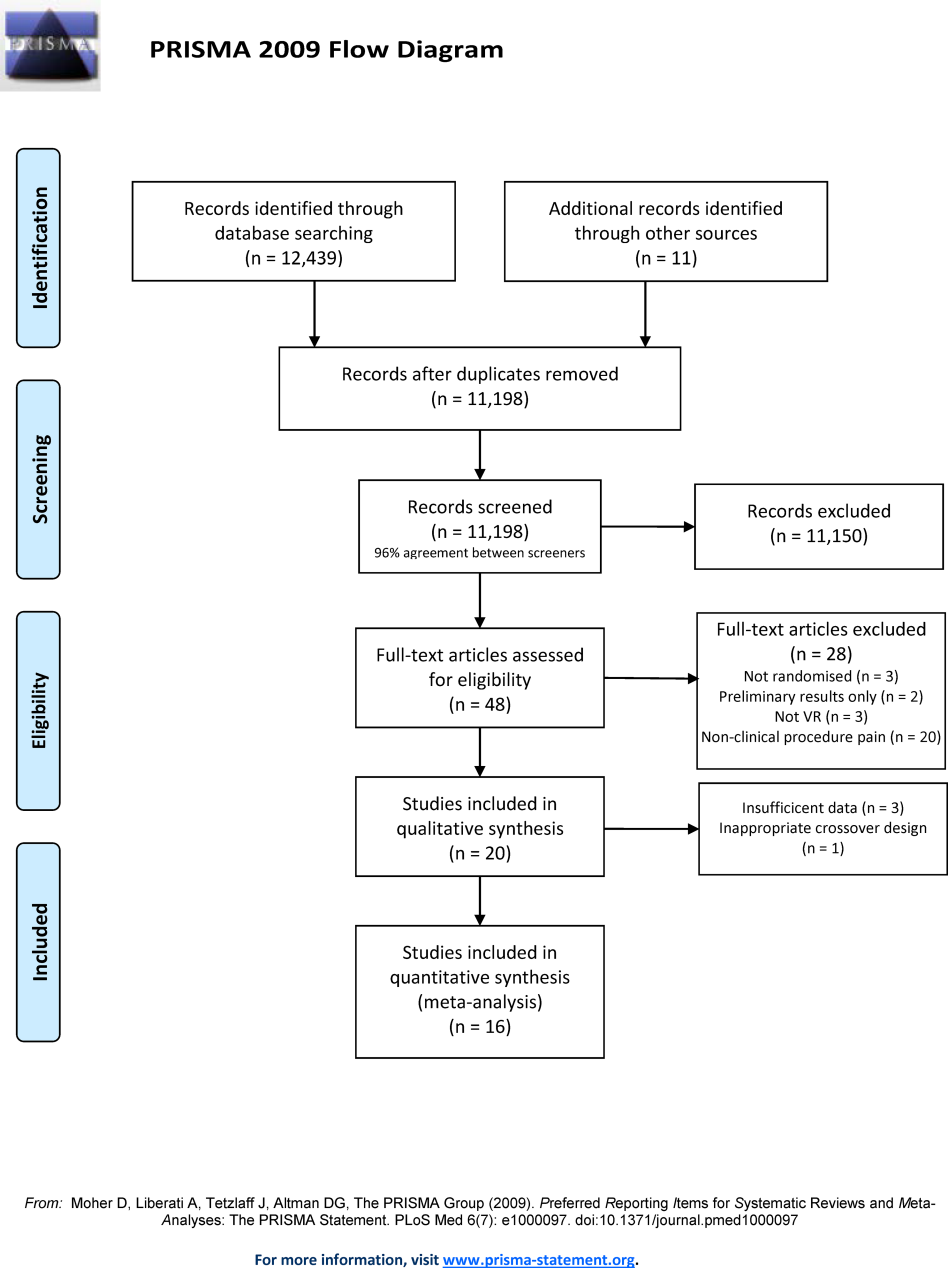
PRISMA diagram.

12,450 studies were screened with 11,150 excluded, leaving 48 full text articles (Fig 1). 28 studies were excluded (predominantly because they examined non-clinical procedures), leaving 20 for qualitative synthesis.

Study characteristics are detailed in Table 1. 11 were RCTs[13–23] and 9 were crossover studies[24–32], studying 776 subjects. 10 studies were performed in the setting of burns wound care[16,18–20,25–29,32], 3 studied physiotherapy in the setting of burns[24,30,31], 5 further studies concerned needle-related procedures (largely venous access)[13–15,21,23], and 2 examined minor surgical procedures[17,22]. Studies were predominantly conducted in English speaking countries (USA (n = 12), Australia (n = 3), South Africa (n = 1)). 11 trials were performed in the inpatient setting, and the remainder were outpatient studies. Pain measurement instruments were heterogenous, but mostly employed 100-point scales.

**Table 1:** Included studies.

Source	Population (age range, years or mean±std dev, females:males)	N	Procedure	Setting	Pain outcome measures	Virtual reality environment, headset type, interaction method	Main findings for VR group versus control group
**Randomised controlled trials**							
Gershon 2004	7–19, 29:30	59	Port access	USA, outpatient	VAS, CHEOPS[33]	*Virtual Gorilla*, interactive game HMD via PC, joystick	No difference in self-rated VAS*, parent VAS or nurse-rated VAS, lower nurse-rated CHEOPS,
Gold 2006	8–12, 8:12	20	Peripheral intravenous cannula	USA, outpatient	FPS-R[34], Wong-Baker FACES[35]	*Street Luge*, interactive game HMD via laptop, inertial tracking,	No difference in child-rated FPS-R* or child-rated Wong-Baker FACES
Gold 2017	10–21, 72:71	143	Venepuncture	USA, outpatient	VAS, CAS, FPS-R	*Bear Blast*, interactive game, HMD-mounted phone, gaze tracking,	After controlling for baseline pain, no difference in self-rated VAS or CAS, but lower self-rated FPS-R*
Guo 2015	18–65, 13:85	98	Hand injury wound care	China, outpatient	VAS	*Afanda*, non-interactive video, HMD via computer	Lower self-rated VAS* after dressing
JahaniShoorab 2015	18–34, 30:0	30	Episiotomy repair	Iran, inpatient	NPRS	*Dolphins and Whales*, non-interactive video, HMD via blu-ray player, gaze-tracking	Lower NRPS* during skin repair (rater not stated)
Jeffs 2014	10–17, 9:19	28	Burns wound care	USA, outpatient	APPT-WGRS[36]	*SnowWorld*, interactive game, HMD via PC, trackball	Lower estimated self-rated APTT-WGRS*
Kipping 2012	11–17, 13:28	41	Burns wound care	Australia, inpatient	VAS, FLACC	*Chicken Little/Need for Speed*, interactive game, HMD via PC, joystick	No difference in adolescent or caregiver reported VAS, but reduction in nurse-rated FLACC at dressing removal^
Konstantatos 2009	18–80, not stated	88	Burns wound care	Australia, inpatient	VAS	*Virtual Medicine*, non-interactive video, HMD via DVD player	Higher self-rated VAS* in VR group
Sander Windt 2002	10–19, 14:16	30	Lumbar puncture	USA, inpatient	VAS	*Escape*, non-interactive video, HMD (PC/DVD not stated)	Lower self-rated VAS^
Walker 2014	18–70, 0:43	43	Rigid cystoscopy	USA, outpatient	VAS	*SnowWorld*, interactive game, HMD (PC not stated), trackball	No difference in self-rated VAS* or proceduralist-rated discomfort VAS
Wolitzky 2005	7–14, 8:12	20	Port access	USA, outpatient	VAS, CHEOPS	*Virtual Gorilla*, interactive game, HMD via PC, joystick	No differences in VAS* (rater unclear), reduction in first-author rated CHEOPS
**Crossover**							
Carrougher 2009	29–57, 4:35	39	Burns physiotherapy	USA, inpatient	GRS	*SnowWorld*, interactive game, HMD (PC not stated), keyboard	Reduction in worst self-rated GRS*
Chan 2007	6.5±2.3, 1:7	8	Burns wound care	Taiwan, inpatient	FACES	*Ice Cream Factory*, interactive game, HMD via PC, mouse	Reduction in self-rated FACES*
Das 2005	5–18, 3:6	9	Burns wound care	Australia, inpatient	FACES	*Custom game*, interactive game, HMD via PC, mouse	Reduction in self-rated FACES^
Hoffman 2008	9–40, 0:11	11	Burns wound care	USA, inpatient	GRS	*SnowWorld*, interactive game, HMD via PC, joystick, interactive	Reduction in self-rated GRS*
Maani 2011	20–27, 0:12	12	Burns wound care	USA, inpatient	GRS	*SnowWorld*, interactive game, HMD via laptop, mouse	Reduction in self-rated GRS*
McSherry 2017	38.4±15.5, 5:13	18	Wound care (various)	USA, inpatient	VNS[37]	*SnowWorld*, interactive game, HMD via laptop, mouse	Reduction in self-rated VNS^
Morris 2010	23–54, 3:8	11	Burns physiotherapy	South Africa, outpatient	NPRS	*Chicken Little*, interactive game, HMD via PC, joystick	Reduction in self-rated NPRS*
Schmitt 2011	6–19, 10:44	54	Burns physiotherapy	USA, inpatient	GRS	*SnowWorld*, interactive game, HMD via laptop, keyboard/mouse	Reduction in self-rated GRS*
Van Twillert 2007	8–65, 7:12	19	Burns wound care	Netherlands, inpatient	VAT	*SnowWorld*, interactive game, HMD (PC not stated), keyboard/mouse	Reduction in self-rated VAT*
**Total n**		776					

VAS, visual analogue scale; CHEOPS, Children's Hospital of Eastern Ontario Pain Scale; FPS-R. Faces Pain Scale Revised; Wong-Baker FACES; CAS, colored analogue scale, NPRS, numeric pain rating scale; APPT-WGRS, adolescent pediatric pain tool word graphic rating scale; GRS, graphical rating scale; VNS, verbal numeric scale; VAT, visual analogue thermometer; HMD, head mounted device; PC, personal computer; DVD, digital video disc.

* denotes meta-analysed outcome.

^ data unavailable for meta-analysis.

10 studies demonstrated high risk of bias in at least 1 domain (Tables 2 and 3). No trials reported in sufficient detail that their risk of bias could be sufficiently assessed across all domains. No trials were prospectively registered and only four studies[17,19,20,31] mentioned CONSORT[38] reporting guidelines. Incomplete reporting or selective reporting was judged at unclear or high risk of bias in 9 studies.

**Table 2:** Bias assessment for randomised controlled trials.

	Randomisation sequence generation	Allocation concealment	Performance bias	Detection bias	Attrition bias	Selective reporting
Gershon 2004	+	?	n/a	?	+	-
Gold 2006	?	?	n/a	?	+	+
Gold 2017	+	+	n/a	?	+	?
Guo 2015	?	?	n/a	?	+	+
JahaniShoorab 2015	?	?	n/a	?	+	+
Jeffs 2014	+	+	n/a	+	+	-
Kipping 2012	+	?	n/a	?	+	+
Konstantatos 2009	+	?	n/a	?	+	=
Sander-Windt 2002	?	?	n/a	?	+	+
Walker 2014	+	?	n/a	?	+	?
Wolitzky 2005	?	?	n/a	?	+	-

Legend:—high risk of bias; + low risk of bias;? unclear risk of bias.

**Table 3:** Bias assessment for crossover trials.

	Appropriate cross over design	Adequate randomisation	Carry-over effect	Unbiased data	Allocation concealment	Detection bias	Performance bias	Incomplete outcome data	Selective outcome reporting
Carrougher 2009	+	?	?	+	?	?	n/a	+	+
Chan 2007	+	+	?	+	?	-	n/a	+	+
Das 2005	-	+	?	+	?	-	n/a	-	?
Hoffman 2008	+	?	?	+	?	?	n/a	+	+
Maani 2011	+	?	?	+	?	-	n/a	+	+
McSherry 2017	+	+	?	+	+	?	n/a	+	+
Morris 2010	+	+	?	+	?	+	n/a	+	+
Schmitt 2011	+	+	?	+	?	-	n/a	-	+
Van Twillert 2007	+	?	?	+	?	?	n/a	-	+

Legend:—high risk of bias; + low risk of bias;? unclear risk of bias.

All trials had short follow up periods and thus attrition bias was generally low. 9/20 studies did not adequately describe their randomisation sequence generation, and 9/11 randomised trials did not describe their allocation concealment in sufficient detail to be assessable.

Data were generally not reported in sufficient detail for detection bias to be assessable, and only one study was assessed at low detection bias risk.

One trial[26] used a crossover design where pain was assessed as being at high risk of being different between baseline and intervention, and was therefore excluded from analysis. No crossover trials specifically reported carry-over effects.

**Fig 2:**
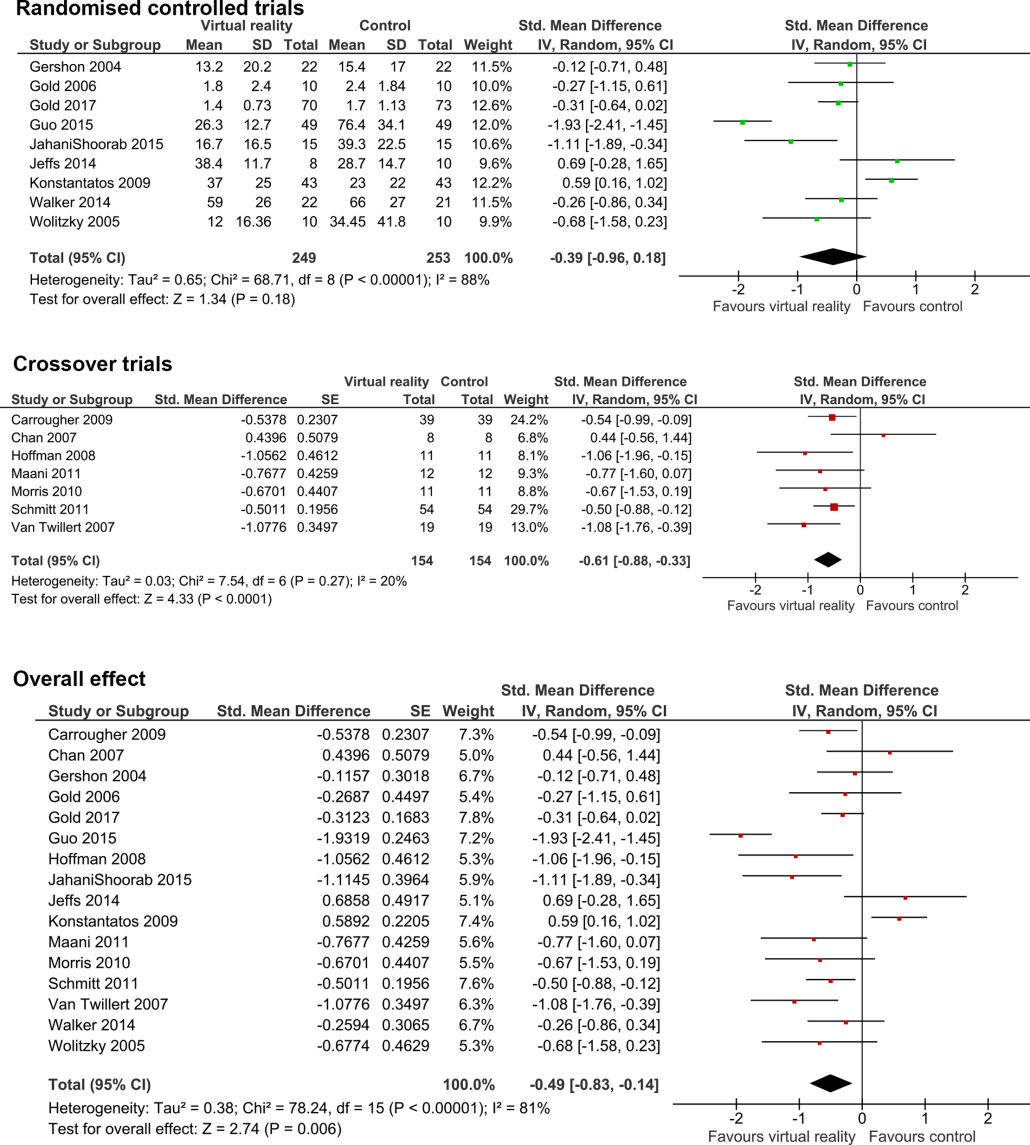
Meta-analysis of the efficacy of virtual reality in acutely painful procedures.

Three further studies were excluded from meta-analysis due to missing data (one group of authors did not respond, one group had destroyed data in accordance with legislation retention requirements, and one group could not provide data due to workload constraints (personal communications)). The meta-analysis therefore consisted of 16 studies for meta-analysis: 9 RCT and 7 crossover, involving 656 individuals (Fig 2).

Statistical heterogeneity[6] was high for RCTs (n = 9, χ^2^ p<0.001, I^2^ 88%, 95%CI for I^2^ 80–93%), low for crossover studies but with a wide confidence interval for I^2^ (n = 6, χ^2^ p = 0.79, I^2^ 20%, 95%CI for I^2^ 0–64%) and considerable overall (n = 16, χ^2^ p<0.001, I^2^ 81%, 95%CI for I^2^ 70–88%). The relatively low number of studies available limited the assessment of the funnel plot., However, no evidence of asymmetry was seen on visual inspection and in particular studies were not absent from the bottom right corner, which would have suggested publication bias (S1 Fig)[12,39].

Meta-analysis of all studies was suggestive of a beneficial effect for VR, with a standardised mean difference pain score reduction of -0.49 (95%CI -0.83 to -0.14, p = 0.006)(Fig 2).

In post-hoc per-procedure subgroup analysis, VR had no effect for minor surgical procedures (SMD -0.65, -1.48 to 0.18, p = 0.13) or burns wound care (SMD -0.46, -1.36 to 0.44, p = 0.31)(S2 Fig). There appeared to be a favourable effect for VR on pain in needles (SMD -0.66, 95%CI -0.56 to -0.04, p = 0.02), and in burns physical therapy (SMD -0.53 95%CI -0.81 to -0.26,p<0.001), although these subgroups enrolled limited numbers of patients (227 and 104 participants respectively).

Statistical heterogeneity assessment was often limited by the relatively few studies present, and reflected in wide I^2^ confidence intervals. For minor surgical procedures (n = 2 studies), some heterogeneity was present (χ^2^ p = 0.09, I^2^ 66%, 95%CI for I^2^ not calculated as too few studies), and for burns wound care (n = 7 studies), there was considerable heterogeneity (χ^2^ p<0.001, I^2^ 92%, 95%CI for I^2^ 85–95%). Though the χ2 test indicated no evidence of heterogeneity for needles (n = 4 studies, χ^2^ p = 0.79, I^2^ = 0%, 95%CI for I^2^ 0–85%) or for burns physical therapy (n = 3 studies, χ^2^ p = 0.94, I^2^ = 0%, 95%CI for I^2^ 0–90%), the confidence intervals for I^2^ were broad.

## Discussion

This systematic review appraises the efficacy of virtual reality for acutely painful clinical procedures, finding that studies were generally at high risk of bias. In meta-analysis, VR appeared to reduce pain in comparison with control, and in post-hoc analysis, the benefit was limited to burns physical therapy and needles.

Applying published, well-accepted criteria, 10/20 studies were at high risk of bias in one or more domain, and no trial reported completely enough for their risk of bias to be completely evaluated. No studies were prospectively registered, and the risk of incomplete or selective outcome reporting was unclear or high in 9 studies. Only four studies reported according to CONSORT guidelines[38].

Meta-analysis indicated a positive effect of VR (SMD -0.49, 95%CI -0.83 to -0.41, p = 0.006) on pain, although the strength of this finding was limited by significant clinical and statistical heterogeneity. Statistical heterogeneity was generally high. This was likely due at least in part to differences in differences in study design and study populations, as well as small study numbers. We chose random-effects meta-analysis to synthesize data in this setting. Although the overall effect may be interpreted by convention as a ‘medium’ effect size[40], benefits appear to differ across different procedural subtypes, with no statistically significant evidence for burns wounds care or minor surgical procedures. Positive effects were driven by needles studies and burns physical therapy studies, raising the possibility that the effect of VR may vary according to study population and clinical scenario. Subgroup analyses were based on small numbers of studies. Importantly, the results of this systematic review and meta analysis are based on less than 1,000 patients in total, with post-hoc subgroup analyses, so findings require confirmation. Before widespread clinical usage of VR can be recommended, large methodologically rigorous studies validating and extending these findings are required.

This study has limitations. VR is a non-blindable intervention that creates methodological issues in bias assessment. Performance bias is un-assessable, and detection bias is difficult to assess, thus we *a priori* defined risk categories. Measures to reduce detection bias can include using independent assessors for study outcomes[6], however, this may be logistically difficult and in paediatric subjects particularly, the patient is at risk of un-blinding the assessor. No crossover studies assessed for carryover effects. However, it seems likely that VR would be reversible and short lived and thus unlikely that VR would have a persistent effect in this clinical context. In addition, study populations were heterogenous, and the precise nature of the hardware and software employed in the VR intervention varied.

We treated VR as a homogenous intervention, although the VR environments and hardware used differed. Even if individual patient data were available, it is unlikely that we would have sufficient statistical power to separate differences between different VR types given significant confounding would exist due to study design, population, and procedure type.

Strengths of our study include a clear clinical question, prospectively registered protocol, thorough search strategy, and the use of high-quality, standardised assessment criteria with more than one assessor at each stage of the review process. We deliberately restricted our selection criteria to clinical studies that were pertinent to our clinical question to maximise external validity. No prior reviews have specifically addressed the clinical question we sought to assess. Existing reviews have not employed a systematic methodology[4], located fewer studies[41], have not performed quantitative data synthesis[42,43], or have focused on special populations[44]. The conclusion of our risk of bias assessment is broadly similar to Garrett[4], inasmuch as we found few trials to be at low risk of bias. The conclusions of our meta-analysis are broadly similar but of a lesser magnitude to Kenney[41], who found a large effect size for VR for painful stimuli in a different group of studies.

## Conclusion

In summary, there is early evidence to suggest that VR is effective for burns physical therapy and needles. However, the quality of the underlying evidence is limited and statistically heterogenous. Thus, prior to widespread adoption of VR, there is a need for further, high-quality studies to validate findings. Trials should be prospectively registered, and reporting should be along CONSORT guidelines to minimise bias. Further studies could include cost-efficacy outcomes, and investigate the role of VR in other acutely painful procedures.

## Supporting information

S1 Checklist PRISMA checklist. (DOC)

S1 Appendix Search strategy. Search executed on 5 November 2017.
(DOCX)

S1 Fig Funnel plot. (EPS)

S2 Fig Post-hoc procedural type meta-analysis. (TIF)
