# Supplementary material for: Clinical efficacy of virtual reality for acute procedural pain management: A systematic review and meta-analysis
Source: PLoS One. 2018 Jul 27;13(7):e0200987. doi: 10.1371/journal.pone.0200987 (PMC6063420; doi:10.1371/journal.pone.0200987)
Supplement: S1 Appendix — Search executed on 5 November 2017. (DOCX) [file pone.0200987.s002.docx]

Search Strategy:

1. Virtual reality exposure therapy/
2. (Virtual or virtuality or VR)
3. (Computer interface or computer simulation)
4. (Simulated or augmented or mediated or mixed) adj3 (reality or world* or environment*))
5. (Head or helmet) adj mounted
6. (Immersi* or Interact*)
7. Distract*
8. OR/1 – 7
9. Exp pain/
10. (Pain* or anesthe* or analges*)
11. Exp fear/
12. (Anxious* or anxiet* or distress* or fear* or worry* or agitat* or apprehensi* or discomfort*)
13. OR/9 – 12
14. 8 AND 13
