# Supplementary figures and images for: Clinical efficacy of virtual reality for acute procedural pain management: A systematic review and meta-analysis
Source: PLoS One. 2018 Jul 27;13(7):e0200987. doi: 10.1371/journal.pone.0200987 (PMC6063420; doi:10.1371/journal.pone.0200987)

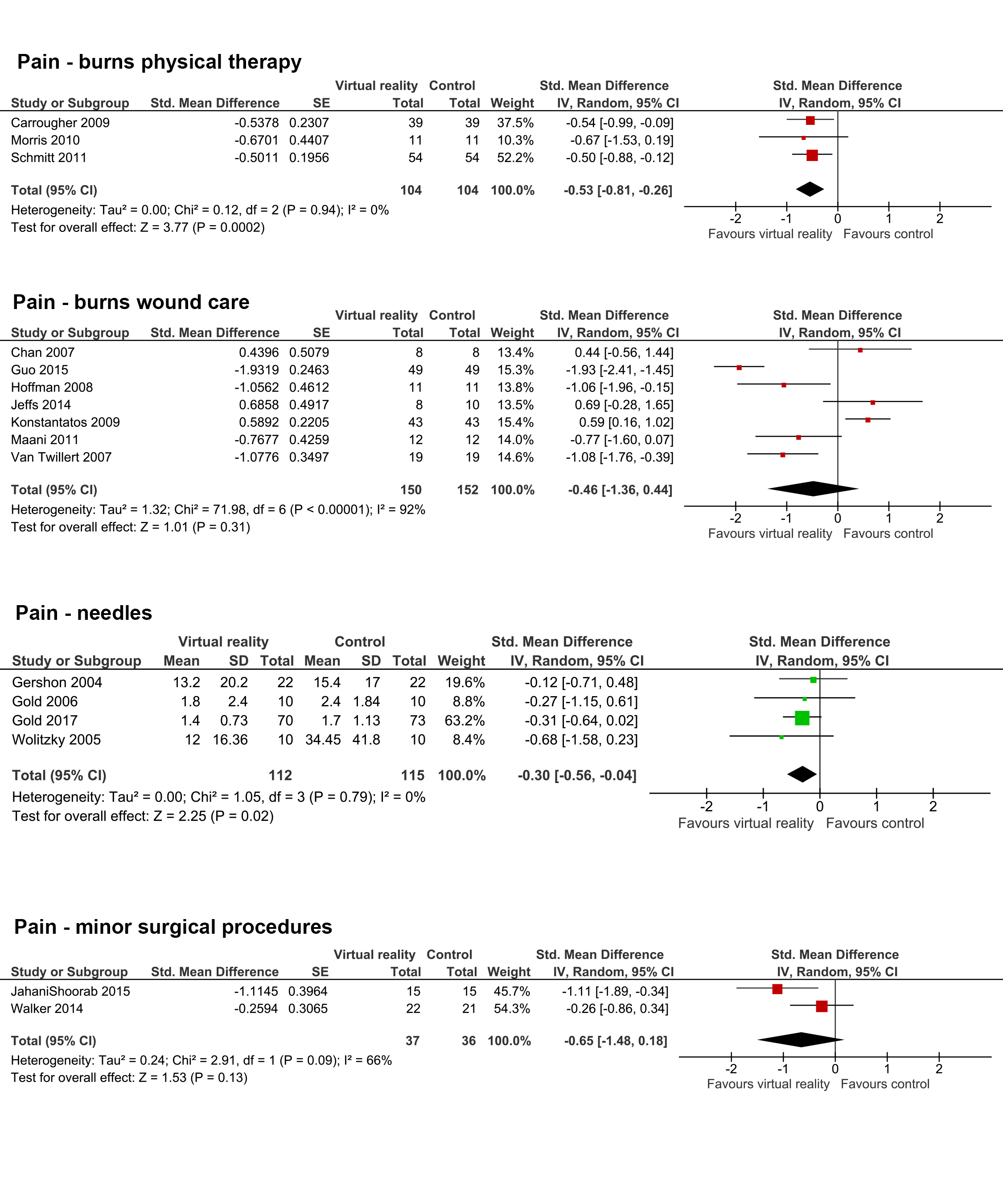

Supplement: S2 Fig — (TIF) [file pone.0200987.s004.tif]
